# Running performance with emphasis on low temperatures in a Patagonian lizard, *Liolaemus lineomaculatus*

**DOI:** 10.1038/s41598-020-71617-3

**Published:** 2020-09-07

**Authors:** N. R. Cecchetto, S. M. Medina, N. R. Ibargüengoytía

**Affiliations:** 1 Ecophysiology and Life History of Reptiles: Research Laboratory, Instituto de Investigaciones en Biodiversidad y Medioambiente, Consejo Nacional de Investigaciones Científicas y Técnicas (INIBIOMA, CONICET-Universidad Nacional del Comahue), 8400 San Carlos de Bariloche, Río Negro Argentina; 2 Centro de Investigación Esquel de Montaña y Estepa Patagónica, Consejo Nacional de Investigaciones Científicas y Técnicas (CIEMEP-CONICET), 9200 Esquel, Chubut Argentina

**Keywords:** Physiology, Ecophysiology, Herpetology

## Abstract

Lizard activity and endurance of cold climate is regulated by several factors such as evolutionary potential, acclimatization capacity, physiological tolerance, and locomotion among thermally advantageous microenvironments. *Liolaemus lineomaculatus*, a lizard inhabiting a wide range of cold environments in Patagonia, provides an excellent model to test interpopulation variability in thermal performance curves (TPCs) and usage of microhabitats. We obtained critical thermal minima and maxima, and performed running trials at eight temperatures using lizards from both a temperate-site (high-altitude) population at 42° S and a cold-site population at 50° S. The availability of environmental temperatures for running performance in open ground and in potential lizard refuges were recorded, and showed that lizards in the temperate site had a greater availability of thermal environments offering temperatures conducive to locomotion. Generalized additive mixed models showed that the two populations displayed TPCs of different shapes in 0.15 m runs at temperatures near their optimal temperature, indicating a difference in thermal sensitivity at high temperatures. However, the rest of the locomotor parameters remained similar between *Liolaemus lineomaculatus* from thermal and ecological extremes of their geographic distribution and this may partly explain their ability to endure a cold climate.

## Introduction

**Figure 1:**
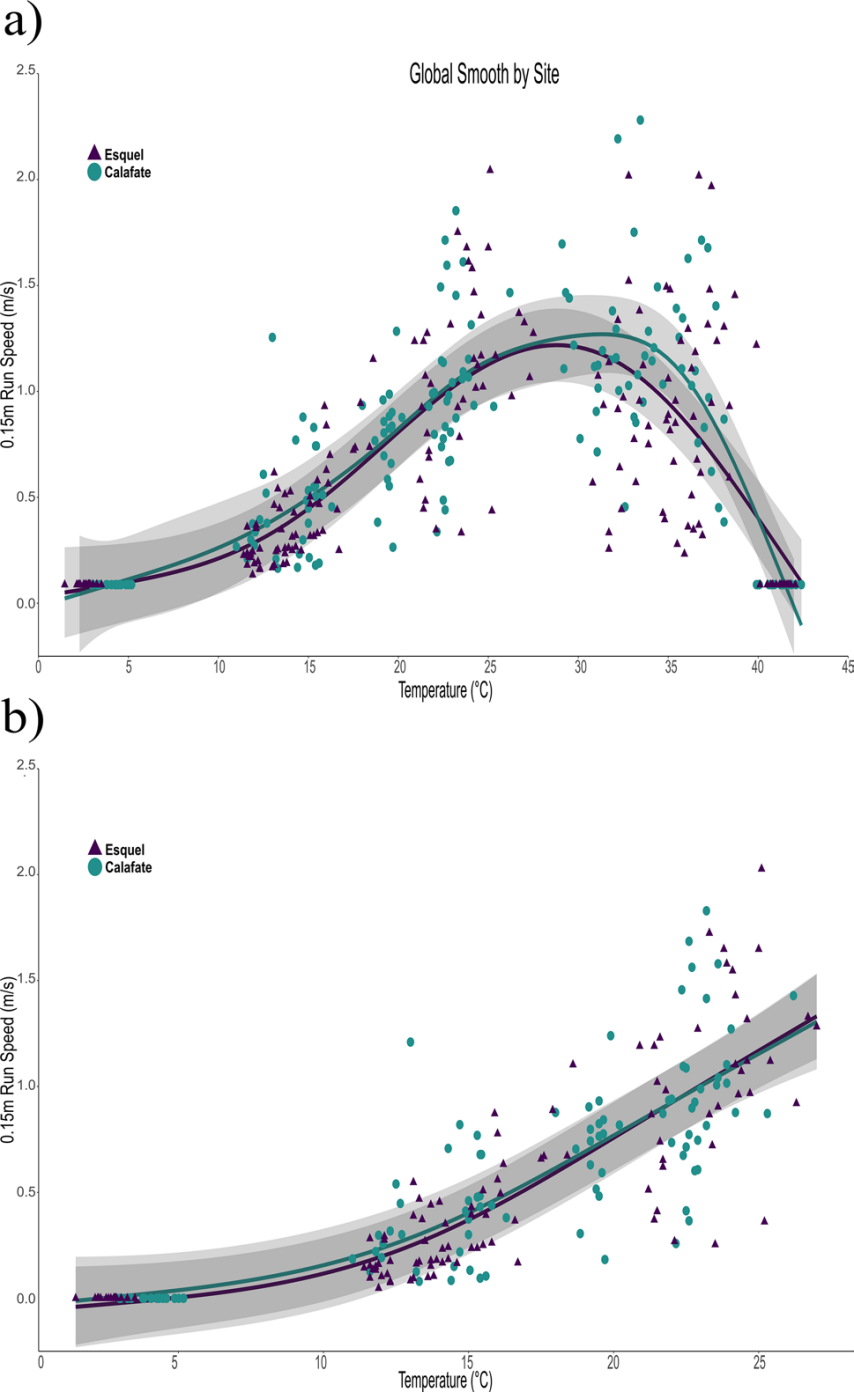
Velocities of 0.15 m runs of *Liolaemus lineomaculatus* individuals from the temperate site (Esquel, triangles) and the cold site (Calafate, circles), and the global smoothing line from the Generalized Additive Mixed Model for each site for (**a**) all temperatures and (**b**) suboptimal temperatures.

**Figure 2:**
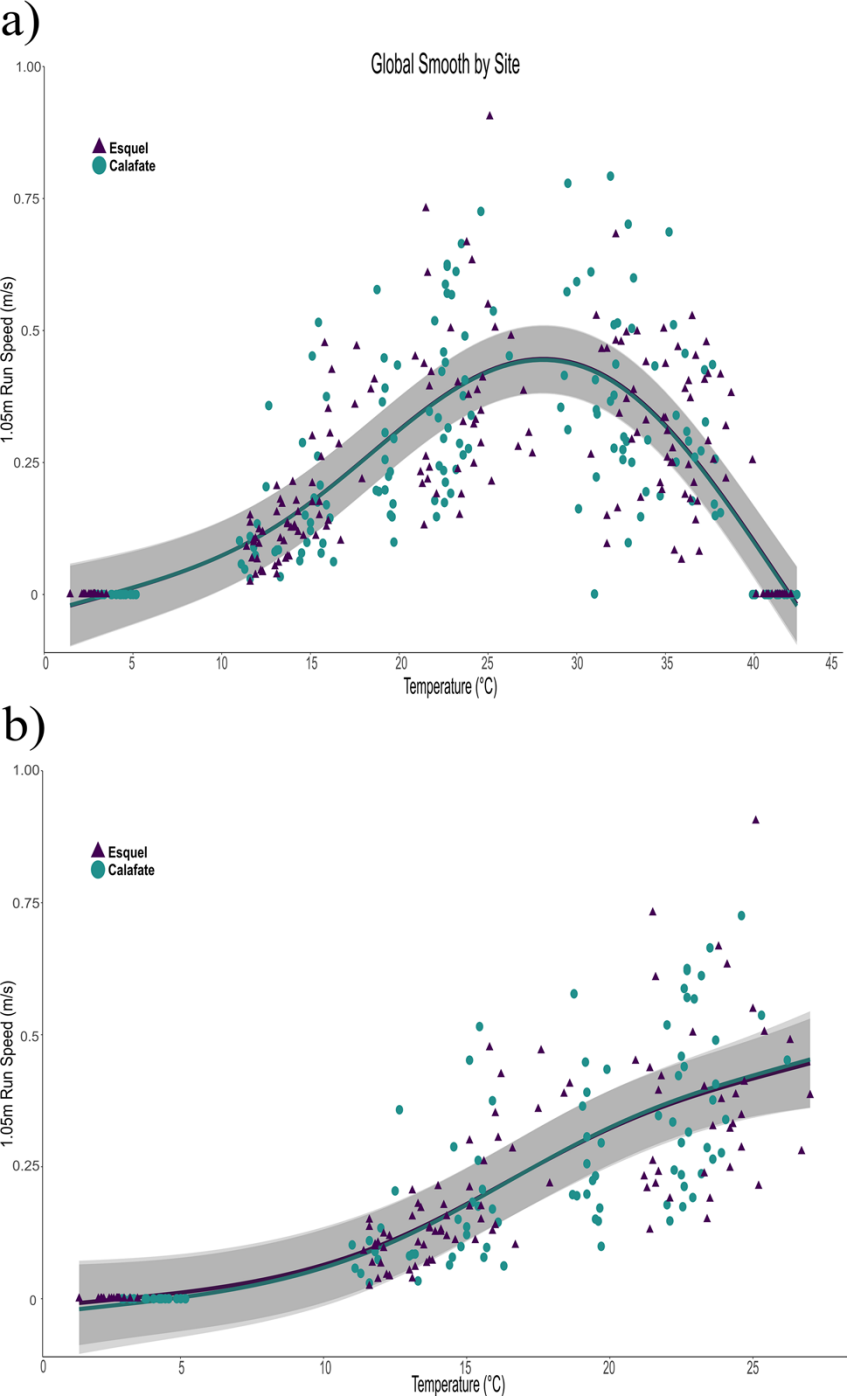
Velocities of 1.05 m runs of *Liolaemus lineomaculatus* individuals from the temperate site (Esquel, triangles) and the cold site (Calafate, circles), and the global smoothing line from the Generalized Additive Mixed Model for each site for (**a**) all temperatures and (**b**) suboptimal temperatures.

In ectotherms, the range of temperatures that allow an individual to roam (thermal tolerance breadth (TTB), sensu Feldmeth et al.^1^) provides an indication of upper and lower limits, outside of which fitness is reduced. For example, individuals may be less able to escape predators, find refuges, or use thermal microenvironments. The TTB for a species restricts the potential hours of activity^2–4^ and is often correlated with its thermal environment^5–8^, varying among populations due to phenotypic plasticity^9^ or natural selection^10^. Within the range of the TTB, the effects of temperature on some performance proxies such as sprint speed, endurance, and digestion, establish the thermal performance curves (TPCs; Figs. 1 and 2). TPCs tend to form a general shape: a sigmoidal increase in performance with temperature, then either a clear peak or a variable plateau at the optimal temperature (*T*_opt_; sensu Waldschmidt and Tracy, Huey and Bennett)^11,12^, depending on the measured performance trait, and finally an exponential or quadratic decrease^13–17^.

The thermal performance curve (TPC) can vary among populations at different locations, given that it is expected that natural selection will favour those phenotypes that maximise performance within their local thermal regime^18–20^. Environmental variability can cause variation in the maximum performance value of the population’s TPC, the *T*_opt_, or the performance breadth (such as 80% or 95% of maximal performance and TTB^16,17,21–23^). Thus, a population’s relationship to temperature can deviate from the species’ average or thermal reaction norm, being best characterized by different mathematical functions (e.g., quadratic, exponential, Gaussian). Low environmental temperatures can be detrimental to vital activities and compromise survival^4,24–26^, unless the population modifies its TPC, its TTB, or makes behavioural adjustments via thermoregulation, modification of the daily hours of activity, or by choosing appropriate refuges to spend inactive time^27,28^.

Lizards from high-elevation or high-latitude environments brumate in winter and, during their behavioural transition during autumn and spring, they frequently experience temperatures near the critical thermal minimum (CTMin). To avoid low temperatures, lizards can choose microenvironments (e.g., burrows, crevices, vegetative cover) where temperatures are warmer than air temperature, and this behavior may extend the hours of activity during transitions. Nevertheless, in temperate and cold environments lizards would still greatly benefit from mechanisms that allow them to be active at low temperatures, even at suboptimal levels of performance, to take advantage of the scant and irregularly available thermal resources in harsh, cold environments. In this regard, lizards can widen their thermal tolerance breadths, modify thermoregulatory behaviour and activity patterns, and be as active at lower body temperatures as are populations in warmer environments^10,29–33^.

The genus *Liolaemus* shows an ability to adapt to a broad range of environments, from Peru, in the northern extreme of their geographic range (12° S), south to Tierra del Fuego, in Argentina (54° S^34,35^), thus providing a very interesting model for testing intraspecific variation in performance. Liolaemids living in the temperate-cold climate of Patagonia showed a remarkable capacity to endure low temperatures, being active at suboptimal temperatures and modifying thermoregulatory behaviour according to the availability of microenvironments for thermoregulation (e.g. *Liolaemus pictus argentinus*^36^,* L. bibronii*,* L. boulengeri*^37^,* L. sarmientoi*, *L. magellanicus*^38^)*.*

Nevertheless, the long period of brumation that reptiles experience in Patagonia in contrast to warmer locations, reduces the hours of activity which in turn affects multiple aspects of their life history^39,40^, and makes it crucial for lizards to find and use the scant warm-temperature resources whenever they are available. *Liolaemus* lizards show slow growth and late sexual maturity (i.e. *L. pictus argentinus*, 6–8 years^41^) in comparison with other Lacertids living in warmer environments^42–44^, and they can adjust their thermoregulation behaviour to compensate for the low environmental temperatures and short periods of activity^37,45,46^. *Liolaemus lineomaculatus* is a viviparous species with a broad distribution from the high-Andean in north-western Patagonia, Argentina, in Neuquén province (39° S), at elevations up to 1,800 m a.s.l., to the lowlands in Santa Cruz province (400 m a.s.l. 51° S^34,35^).

In this study, we evaluated the locomotor performance of *Liolaemus lineomaculatus* in laboratory trials at several temperatures, with emphasis on the low-temperature portion of the thermal tolerance breadth. We selected two populations located at the extremes of the species eco-geographic range: a northern one in the high-Andean steppe, at 1,800 m a.s.l. in Esquel (42° S), and a southern one in the lowland steppe, in Calafate (50° S), Argentina. Results of the thermal performance of *Liolaemus lineomaculatus* are discussed in relation to the ecological implications of locomotor capacities at low temperatures (near CTMin) in harsh environments of Patagonia.

Given that Patagonian *Liolaemus lineomaculatus* populations are living in the extremes of the species distribution, we hypothesize that:

1. Patagonian lizard populations live in environments of relatively different “thermal quality” (i.e., microhabitats with different ecologically relevant temperatures for the species, sensu Huey^47^).
  From this hypothesis, we predict wider variability of thermal microenvironments with temperatures within thermal parameters of eco-physiological relevance (thermal optima or thermal tolerance breadths for running performance) at the high-elevation site in Esquel than at the lowlands in Calafate, probably affecting the hours of activity in both populations.
2. The individuals from these two populations have adapted their locomotor performance capacities, particularly at suboptimal temperatures, and different thermal sensitivities, according to the thermal quality of the environment. From this hypothesis, we predict that lizards from the population with low thermal quality will run at higher speed at suboptimal temperatures than the population that inhabits the environment with higher thermal quality. Additionally, we predict that the shapes of the thermal performance curves of these two populations will be different, indicative of different sensitivities to temperature (wider or narrower thermal performance breaths, different maximum speeds, or different slopes).

## Results

### Parameters of the thermal performance curves for the 0.15 m runs and the 1.05 m runs for lizards from the temperate population (Esquel) and from the cold population (Calafate)

Thermal tolerance breadth (TTB) was wider (Table 1) and notably CTMin was lower (*t*_*1,27*_ = 7.27, *p* < 0.01) in lizards from the temperate-site population (Esquel, mean = 2.67 ± 0.48) than in lizards from the cold-site population (Calafate, mean = 4.18 ± 0.72). There was no significant population difference in CTMax (*t*_*1,27*_ = 0.98, *p* = 0.33).

**Table 1:** Temperature range for *Liolaemus lineomaculatus* from the temperate (Esquel) and the cold (Calafate) populations’ locomotor performance parameters.

Population parameter	Temperature range (°C)			
	Esquel		Calafate	
	Lower bound	Upper bound	Lower bound	Upper bound
Thermal tolerance breadth	1.46	42.06	2.97	42.40
**0.15 m runs**				
B_80_ range	24.43	35.09	23.70	34.46
B_95_ range	27.71	32.63	26.89	31.93
**1.05 m runs**				
B_80_ range	21.15	34.68	21.29	34.83
B_95_ range	24.84	31.40	25.67	31.65

Thermal tolerance breadth represents the difference between CTMax and CTMin, while the B_80_ and B_95_ ranges are the ranges of temperatures within which the populations can achieve 80 and 95% of their maximum speed, respectively.

For both types of runs, we calculated the performance breadth as the ranges of T_b_ at which performance is greater than or equal to 80% and 95% of maximum speed, respectively (B_80_ and B_95_). For the 0.15 m runs (Fig. 1a), the higher and lower bounds of B_80_ were significantly higher for individuals from the temperate site (mean_lower bound_ = 24.43 ± 0.21; mean_higher bound_ = 35.10 ± 0.26) compared to individuals from the cold site (mean_lower bound_ = 23.70 ± 0.38; mean_higher bound_ = 34.50 ± 0.30; *t*-test, *t*_*1,36* lower bound_ = 7.73*, p* < 0.01; and *t*_*1,36* higher bound_ = 8.73*, p* < 0.01). There were no significant differences in maximum speed (V_max_), maximum speed at suboptimal temperatures (V_suboptimal_), or optimal temperature (*T*_opt_) between populations (*F*_*1,36* Vmax_ = 0.23,* p* = 0.64; *F*_*1,34 Vsuboptimal*_ = 1.89, *p* = 0.18; and *F*_*1,36 Topt*_ = 0.65,* p* = 0.43). For the 1.05 m runs (Fig. 2a), individuals from the temperate site (mean_lower bound_ = 21.80 ± 0.51) showed lower values for the lower bound of B_80_ (*t*_*1,36* lower bound_ = 4.59, *p* < 0.01) than individuals from the cold site (mean_lower bound_ = 22.60 ± 0.60). There were no differences in the upper bound of B_80_, nor in V_max_, V_suboptimal_ or *T*_opt_ between populations (Table 2). Individual performance curves for 0.15 m and 1.05 m runs are in the Supplementary Information section (Supplementary Figs. 2–5).

**Table 2:** Comparison of mean performance parameters of 0.15 m and 1.05 m runs, and critical thermal minima and maxima (°C) including the lower and upper values of the performance breadth (B_80_ lower and B_80_ upper, °C), maximum speed (V_max_, m/s), maximum speed at suboptimal temperatures (V_max suboptimal_, m/s), and thermal optimum (T_opt_, °C).

Population parameter	Esquel mean	Calafate mean	Statistic	*p*
CTMin	2.67	4.18	*T*_1,27_ = 7.27	**< 0.01**
CTMax	41.1	41.3	*T*_1,27_ = 0.98	0.33
**0.15 m runs**				
B_80 upper_	35.1	34.5	*T*_1,16_ = 8.73	**< 0.01**
B_80 lower_	24.4	23.7	*T*_1,16_ = 7.73	**< 0.01**
V_max_	1.41	1.74	*F*_1,36_ = 0.23	0.64
V_suboptimal_	1.27	1.10	*F*_1,34_ = 1.89	0.18
*T*_opt_	30.17	29.66	*F*_1,36_ = 0.65	0.43
**1.05 m runs**				
B_80 upper_	33.99	34.18	*T*_1,16_ = 0.13	0.89
B_80 lower_	21.81	22.65	*T*_1,16_ = 4.59	**< 0.01**
V_max_	0.52	0.63	*F*_1,36_ = 3.46	0.07
V_suboptimal_	0.45	0.44	*F*_1,34_ = 0.05	0.83
*T*_opt_	28.12	28.86	*F*_1,36_ = 1.16	0.22

Statistical parameters for t-tests (*T*), Fischer’s test (*F*), and probabilities (*p*) are shown. Performance parameters were obtained as the means of the estimates of each individual thermal performance curve. Bold letters indicate significance values of *p* < 0.01.

### Proportion of individuals running within B_80_ and B_95_ in the 0.15 m and the 1.05 m runs

A higher proportion of lizards from the temperate population (Esquel) than lizards from the cold population (Calafate) ran at speeds above their respective B_80_ and B_95_ parameters, in the 0.15 m runs, while for the 1.05 m runs no significant population differences were found.

For the 0.15 m runs, 86% of individuals from the temperate site (18 of 21) and 53% of individuals from the cold site (9 of 17) ran at a speed within the B_80_ (Fisher’s exact test; odds ratio = 5.08, p = 0.03). Furthermore, 62% of individuals from the temperate site (13 of 21) and 29% of individuals (5 of 17) from the cold site ran at a speed within the B_95_ (Fisher’s exact test; odds ratio = 3.75, p = 0.04).

For the 1.05 m runs, 67% of individuals from the temperate site (14 of 21) and 59% of individuals from the cold site (10 of 17) ran at a speed within the B_80_ (Fisher’s exact test; odds ratio = 1.39, p = 0.43). Additionally, 52% of individuals from the temperate site (11 of 21) and 41% of individuals from the cold site (7 of 17) ran at a speed within the B_95_ (Fisher’s exact test; odds ratio = 1.55, p = 0.36).

### Models testing and comparison of the thermal performance curves (TPC) between populations

An AIC comparison of the models with and without “individual” as a random effect showed a significant improvement when including the random effect in the 0.15 m and the 1.05 m runs models (Supplementary Information section, Supplementary Table).

The GAMMs fits on the TPC showed a significant effect of the smoothing term on temperature (F_1,7.33_ = 43.9, p < 0.01 for the 0.15 m runs and F_1,6.76_ = 84.3, p < 0.01 for the 1.05 m runs), and significantly different trends in 0.15 m run between individuals from the temperate site and the cold site (F_1,4.29_ = 2.54, p = 0.03, Fig. 1a). In the 1.05 m runs, we did not find a significant difference in shape between the TPCs (Fig. 2a). The random effect of “individuals” was significant for both models (F_1,24.14_ = 2.72, p < 0.01 for the 0.15 m run and F_1,27.58_ = 5.15, p < 0.01 for the 1.05 m runs), and the covariables BCI and sex did not have significant effects on any of the models. Deviance explained by the 0.15 m run model was 73.3%, while the 1.05 m runs model explained 74.1% of deviance.

Meanwhile, the GAMM fits for the suboptimal temperatures TPC (i.e. below *T*_opt_) showed a significant effect of the smoothing term on temperature (F_1,2.66_ = 131.03, p < 0.01 for 0.15 m runs, and F_1,2.73_ = 96.56, p < 0.01 for the 1.05 m runs), but the model did not detect a significant difference in shape between the TPCs in 0.15 m runs or 1.05 m runs (Figs. 1b, 2b). The random effect of “individuals” was again significant in both models (F_1,23.97_ = 2.63, p < 0.01 for 0.15 m runs and F_1,26.28_ = 3.96, p < 0.01 for the 1.05 m runs), and the covariables BCI and sex did not have a significant effect on any of these models either. Deviance explained by the 0.15 m runs model was 81%, while the 1.05 m runs model explained 80% of deviance (Table 3).

**Table 3:** Generalized additive models (GAMs) fit to sprint-runs and long-runs, in individuals from Esquel (temperate site) and Calafate (cold site).

	Estimation of parametric coefficients (*SE*)		Approximate significance of the elevation smoothing term (s) and interactions						Deviance explained (N)
	Intercept Esquel	Intercept Calafate	s (temperature)		s (temperature:Calafate)		s (individual)		
			*F*-value (edf)	*p*	*F*-value (edf)	*p*	*F*-value (edf)	*p*	
0.15 m runs	**0.49** (0.15)	0.43 (0.17)	43.9 (7.33)	**< 0.01**	2.54 (4.29)	**0.03**	2.72 (24.14)	** < 0.01**	73.3% (358)
1.05 m runs	**0.16** (0.07)	0.07 (0.07)	84.3 (6.76)	**< 0.01**	0.01 (1)	0.95	5.15 (27.58)	**< 0.01**	74.1% (356)
0.15 m runs at Suboptimal temperatures	**0.48 **(0.15)	0.45 (0.17)	131.03 (2.66)	**< 0.01**	0.46 (1.33)	0.69	2.63 (23.97)	**< 0.01**	81% (213)
1.05 m runs at Suboptimal temperatures	0.09 (0.07)	− 0.06 (0.08)	96.55 (2.73)	**< 0.01**	0.16 (1)	0.69	3.96 (26.28)	**< 0.01**	80% (212)

For each thermal performance curve (TPC), the parametric coefficients are the intercepts of the models estimated for each population. An Analysis of Variance (ANOVA) with an F-test was used to evaluate changes in the shape of TPC between populations, for the 0.15 m runs and for the 1.05 m runs. *SE* standard error, *N* number of observations, *edf* effective degrees of freedom. Bold letters indicate significance values of *p* < 0.01.

### Environmental temperatures and its relationship with running performance in *Liolaemus lineomaculatus*

**Figure 3:**
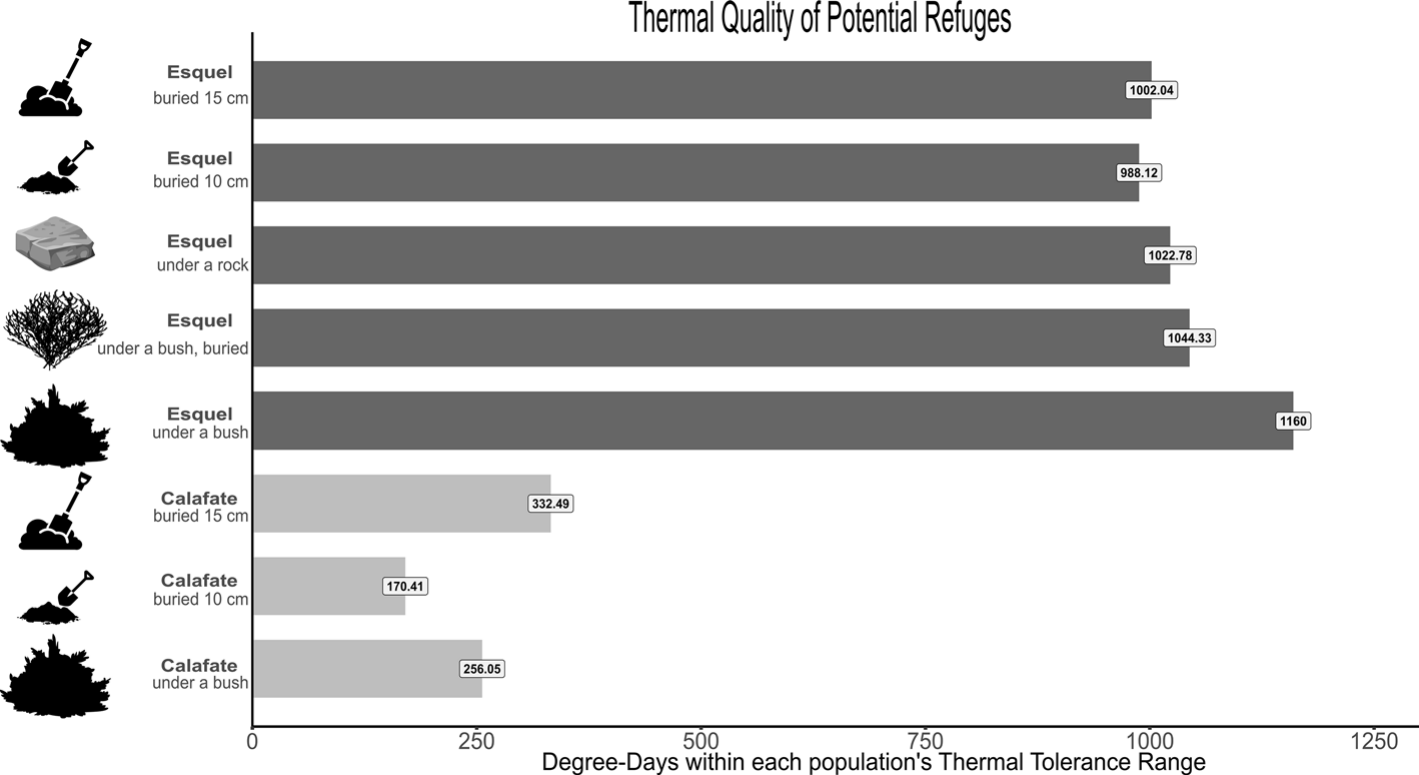
Thermal quality of the potential refuges (degree-day) in the temperate site (Esquel, dark grey) and the cold site (Calafate, light grey). Values for degree-days within each population’s thermal tolerance breadth (TTB) are represented for each potential refuge. Vector art obtained or modified from https://svgsilh.com; https://pixabay.com; https://www.cleanpng.com.

The environmental temperatures recorded by data-loggers obtained from the PVC lizard models of potential overwintering refuges and exposed microenvironments on the ground at each sampling site showed that, in the temperate site (Esquel), lizards can spend longer time at favourable temperatures for running performance than in the cold site (Calafate). Lizards in the temperate site have longer time of availability of environmental temperatures within the thermal tolerance breadth (TTB), the B_80_, the B_95,_ and longer time to attain the *T*_opt_, than lizards from the cold site (Table 4). Degree-days within TTB were almost four times higher for the potential refuges in the temperate site than for potential refuges in the cold site (Fig. 3).

**Table 4:** Hours of activity spent within the range of the locomotor performance parameters for each population and the percentage of the total hours of activity they represent.

Active time (hours) spent in the range (percentage of total)		
Population parameter	Esquel	Calafate
Thermal tolerance breadth	2,262 (95%)	1,693 (71%)
**0.15 m runs**		
B_80_ range	329 (14%)	123 (5%)
B_95_ range	135 (6%)	51 (2%)
*T*_opt_	28 (1%)	11 (1%)
**1.05 m runs**		
B_80_ range	615 (26%)	188 (8%)
B_95_ range	243 (10%)	67 (3%)
*T*_opt_	44 (2%)	11 (1%)
**Total**	2,378

## Discussion

Despite the high elevation, the population of *Liolaemus lineomaculatus* at Esquel (temperate site) experiences more degree-days at optimal locomotor performance temperatures than the population living in the cold site, in Calafate, particularly during spring and autumn. Lizards in Esquel experience more of their activity span at temperatures within their thermal tolerance breadth than lizards in Calafate. In particular, during the coldest seasons when lizards are starting or finishing brumation and still in intermittent activity (autumn and spring) the degree-days at the potential refuges were four times higher in the temperate site than in the cold site. We found that these environmental differences are associated with changes in sensitivity to temperature, represented by a difference in thermal tolerance breadth and a different shape of the thermal performance curves in the 0.15 m runs. While both populations show exponential decreases for values above optimal temperature, the population from the cold site has a steeper exponential drop for values above ~ 30 °C (T_opt_) for 0.15 m runs and for values above ~ 28 °C (T_opt_) for 1.05 m runs in comparison with the population from the temperate site*.*

**Figure 4:**
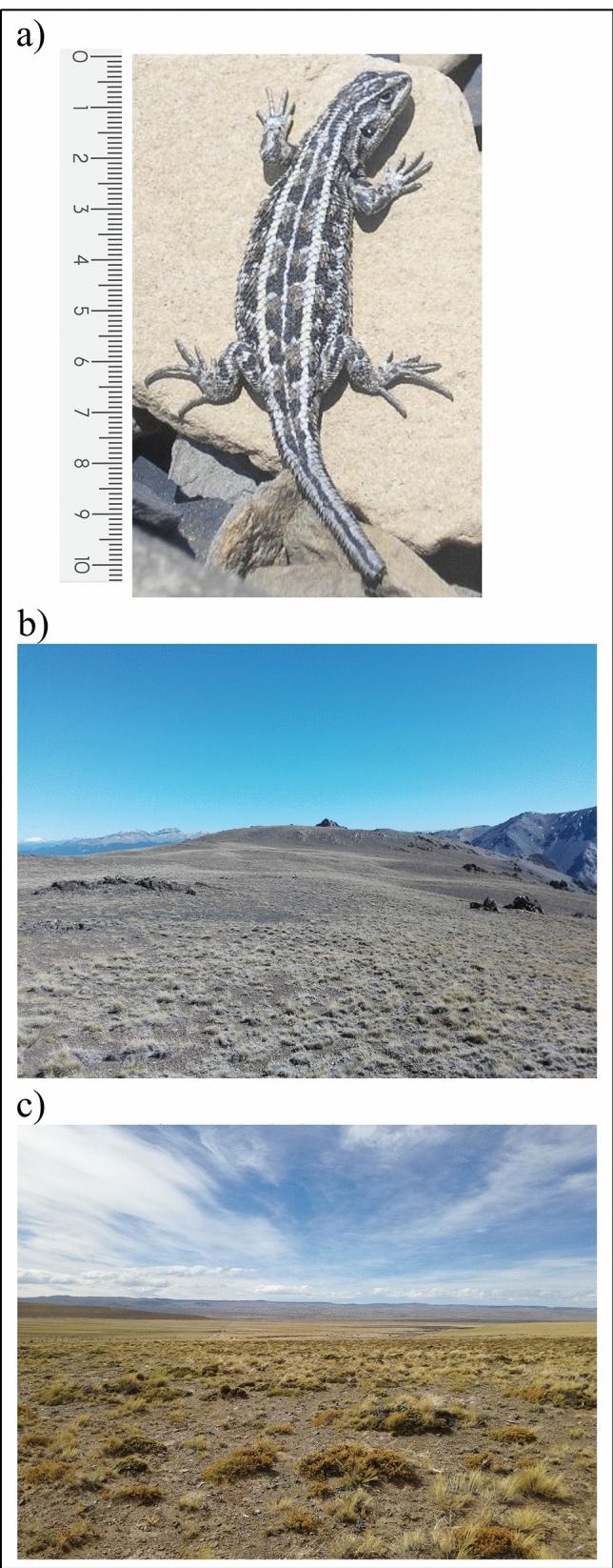
(**a**) A photograph of a *Liolaemus lineomaculatus* individual, scale in cm. (**b**) A photograph of the sampling site in Esquel (temperate site). (**c**) A photograph of the sampling site in Calafate (cold site).

The thermal tolerance breadths for individuals from Esquel were wider, with lower critical thermal minimums than for individuals from Calafate. The lower bound of B_80_ for the 1.05 m runs was almost 1 °C lower in lizards from Esquel as well. This is not surprising, since many studies show that CTMin can vary across latitudes and elevations for many terrestrial ectotherms^33,48^. However, the lower and upper bounds of B_80_ for the 0.15 m runs was almost 1 °C lower for individuals from the cold site than for individuals from the temperate site. This difference suggests an adaptive shift or plasticity of the performance curve to colder temperatures in Calafate, which would allow lizards living in a harsher environment to perform at the same speed at lower temperatures. However, although this potential advantage was observed in 0.15 m runs, there were no differences when lizards had to run longer distances (1.05 m runs). The great importance of sprint speed for many ectotherms’ fitness and survival is evident in events such as fleeing predators^49,50^ and capturing prey^51^. Therefore, it is not surprising that the 0.15 m run speed might have population-level differences in thermal sensitivities in comparison with other locomotor parameters such as the 1.05 m run speed. This difference in thermal sensitivity might also be explained by ecological factors such as a difference in predation pressure^52,53^ or differences in the landscape and type of substrate used for most vital activities such as feeding, reproduction and exploration. For example, the high-Andean steppes in Esquel feature small areas of variable steepness between potential refuges and irregular distances between refuges, a characteristic not present in the steppes of Calafate, which are mostly open plains with more-uniform distances between shrubs (Fig. 4b,c).

In the field, we found several differences in the thermal quality of the environments exposed (out of potential refuges) and in the thermal quality of the potential refuges that *Liolaemus lineomaculatus* could use in the intermittent and opportunistic activities during the hours of activity in autumn and spring. In the high-Andean steppes from the temperate site, in Esquel, lizards spent the majority (95%) of autumn, spring and the beginning of summer within their thermal tolerance breadth (TTB). In contrast, in the steppes of Calafate, the cold site, lizards spent only 71% of activity time during the same months within their TTB. The same pattern can be observed for the B_80_ and B_95_ ranges and for *T*_opt_ in both the 0.15 m runs and the 1.05 m runs. Therefore, Esquel lizards might inhabit an environment that provides a better thermal quality for running performance. It should be noted that the sampling of potential refuges did not take into account relative frequency of all available potential refuges nor were we able to deploy enough models to obtain replicas of each potential refuge at each site, so certain types of refuges might be overrepresented and others underrepresented. Nevertheless, the homogeneity of the environment allowed us to cover the most representative microenvironments even with few models (Fig. 4b,c). A more extensive study with more models per site, as recommended by some authors^54^ would be necessary to describe more accurately their thermal environments.

The variance in thermal quality and physiognomy of the landscape did not result in differences in maximal velocities of the 0.15 m runs or the 1.05 m runs between populations. We expected the lower thermal quality of partially exposed and potential refuges models in the cold site to be correlated with a better running performance by those individuals, to compensate for having less time available with temperatures within the TTB, as seen in many terrestrial ectotherms such as insects, amphibians and reptiles^55^. Additionally, daily temperature amplitude is more variable at high elevations and, when daily variation is situated near the most thermally sensitive areas of the TPC (such as values near *T*_opt_ or near the critical thermal minima or maxima), it can reduce performance^56^, which could affect individuals from the temperate site. Nevertheless, none of these factors seems to be correlated with differences in maximal velocities between the populations. Physiological limitations, such as mechanical power output of the muscle fibres in relation to temperature^26,57^, might be favouring conservation in speed-related traits such as V_max_ despite environmental differences.

In spite of the mentioned differences in the thermal quality of the environments, we did not detect significant differences in the optimal temperature between populations, even though optimal temperatures for running are considered to be lower in lizards in colder temperate environments^24,38^. Mean optimal temperatures of liolaemids seem variable among species, particularly in lizards of the *lineomaculatus* section (from 27 to 36 °C^38,58^). However, we found that between populations of *Liolaemus lineomaculatus* in different environments, *T*_*opt*_ for the 0.15 m and the 1.05 m runs remains consistent and within the range of values found for other liolaemids^58,59^. Some of the factors that could be keeping optimal temperatures similar among populations within a species, as is the case for *Zootoca vivipara*^25^ and *Sceloporus undulatus*^60^, are behavioural adjustments such as thermoregulation^61^ and microhabitat selection^62^. Additionally, optimal temperatures could be similar among populations within a species because of changes in predation strategies, or because of differences in selection pressure at the different locations that maintain the optimal temperature at a similar value, as was proposed by van Damme^25^. Values of both populations *T*_*opt*_ are below *Liolaemus lineomaculatus’* preferred laboratory temperatures (T_sel_)^63^, as is the case for *L. pictus argentinus*^59^, *L. sarmientoi and L. magellanicus*^38^, and the gecko *Homonota darwini*^64^. Patagonian lizards are able to obtain maximal performance output even below preferred laboratory temperatures, which could be another cold-environment adaptation in the suite of traits composing their life histories typified by late sexual maturity, longevity, and low mean annual reproductive output^65,66^.

The Generalized Additive Mixed-effects Models showed that the mixed structure, considering individuals as a random effect, significantly improved all models. Interindividual variation in the populations’ life history traits has been proved to be an important source of variability^67,68^, which could have key relevance in the species’ plasticity, expansion and distribution^69^, and is sometimes more important than interpopulation variability^70^. We provide further evidence that studies of thermal performance curves should include interindividual variability while modelling for population trends with a statistical model that contemplates this very complex structure of individuals with variable tendencies.

The GAM approach allowed us to see some marginal differences in the shape of the TPC between individuals from the temperate site, Esquel, and those from the cold site, Calafate, in the 0.15 m runs (Fig. 1a), but we did not find differences in the 1.05 m runs (Figs. 1b, 2a,b). This is interesting because even though traditionally it has been considered that TPCs tend to take the same general shape^13,71^, there seems to be value in allowing the model to consider population-specific shapes and allowing for variability per individual (see the Supplementary Information section for individual performance curves, Supplementary Figs. 2–5). However, that for some species thermal physiology is evolutionarily conservative and thus relatively insensitive to directional selection, following the “static thermoregulation view” (sensu Hertz et al.^72^), such as *Psammodromus algirus*, where high-elevation lizards did not perform better than mid- and low-elevation lizards at suboptimal body temperatures, despite inhabiting a low-quality thermal environment^73^.

Lizards in Esquel seem able to attain more of their locomotor potential than lizards in Calafate, since a higher proportion of the population ran at speeds above the B_80_ and B_95_ parameters in the 0.15 m runs. Perhaps this is due to living in a more heterogeneous environment with better opportunities for thermoregulation, as seen by the potential refuges analysis^74–77^.

Evidence suggests that the state of the surrounding environment can have a profound effect on the perception of “fear” by prey animals in predatory encounters; there is a strong effect of distance to the refuge in most species, and more species-specific evidence of effects of group size, habitat type and patch quality^78^. In the foraging literature, the environmental stochasticity (in this case, considering the *temperature* resource) is usually referred to as “risk”, and the daily energy budget rule^79^ states that a forager on a positive budget should be risk-averse while a forager on a negative budget, risk-prone^80^. Following this logic, if lizards from the cold site in Calafate were living on a negative thermal budget, they would be more risk-prone in comparison to lizards from the temperate site in Esquel. In Calafate, lizards might be forced to leave their refuges to thermoregulate in risky situations where speed might factor in their survival^81^, making speed an important trait to develop. Meanwhile, the potential refuges in Esquel might allow lizards to avoid unnecessary risks since they showed four times the amount of degree-days in the thermal tolerance breadth in comparison to potential refuges in Calafate, providing the lizards enough temperature to move without having to leave the refuge (Fig. 3). Additionally, the high-Andean steppes in Esquel provide more variability in types of microsites to use as provisional refuges, such as rocks and burrows dug by small mammals, absent in the steppes in Calafate. Microsite selection might play a larger role than mean ambient temperature or even latitude in shaping TPC parameters^8^. Therefore, this difference in potential refuges may be even more important than the difference in temperature observed between exposed model temperatures, especially since presence or vulnerability to predation might act against continuous activity even during favourable weather^53,82^.

In the cold weather and great seasonal thermal variations of Patagonia, at the high elevation of the Andean steppes of Esquel and in the southern latitude steppes of Calafate, *Liolaemus lineomaculatus* manages to survive and display an array of behaviours related to temperature and locomotion. In our study, we have seen that *L. lineomaculatus* is able to function at environments of different thermal quality with similar performance. Regarding 0.15 m runs, the species modified the shape of their thermal performance curves between populations, and there was a shift to colder temperatures in the population from Calafate. No such changes were found regarding 1.05 m runs, or considering only temperatures below *T*_*opt*_*.* Future studies could inquire into the genetic component that explains this interindividual variability in performance and the variability among populations of a same species in relatively similar environments with common garden experiments or translocations, to differentiate between adaptation and plasticity. Future studies could also investigate the characteristics of potential refuges based on behavioural observations in the field and on the use of tracking technology to disclose which refuges lizards actually use in the field, particularly during winter.

## Materials and methods

### Study areas and field methods

*Liolaemus lineomaculatus* is a small (SVL = 62 mm; Fig. 4a), insectivorous, psammophilous, viviparous lizard^34,35^. We captured adults at two extreme locations of the species’ eco-geographic range: one in the Andes near Esquel, Argentina (42° 49′ S, 71° 15′ W; 1,800 m a.s.l.; March 2017; N = 21, 13 males and 8 females, Fig. 4b), and the other in the steppes of Calafate (50° 15′ S, 71° 29′ W; 450 m a.s.l.; February 2018, N = 17, 7 males and 10 females, Fig. 4c). We captured lizards by hand or loop, and individuals were handled by the head and hips at time of capture to avoid heat transfer.

In the high-Andean steppe, lizards can find refuge under boulders, bushes, tussocks or in the many abandoned burrows of small mammals (such as rodents from the *Ctenomys* genera), and the terrain is composed of small areas of variable steepness. Meanwhile, in the steppes near Calafate, the terrain is a plain, open field with numerous bushes and tussocks, but there are almost no boulders or rocks to hide under or use as heat sources (N. Cecchetto, *personal observation*).

### Effects of body temperature on speed

Immediately after capture, we brought lizards to the laboratory in individual cloth bags to minimize stress, and housed them in individual open-top terraria (15 × 20 × 20 cm). We carried out the locomotor performance trials (running trials) within 96 h of capture between 09:00 and 19:00 h, when lizards are active in their natural environment and at least 16 h after feeding. Lizards were fed and had water ad libitum daily after completing the trials.

Running trials were conducted on a racetrack 0.07 m wide and leading to a shelter. Eight photocells positioned at 0.15-m intervals along the track and connected to a computer sensed the lizard’s motion, and thereby, the speed over each 0.15-m section and the full 1.05 m length. During analysis, each run was broken into a sprint-run component (first 0.15 m, henceforth referred to as “0.15 m run”), and a long-run component (henceforth referred to as “1.05 m run”), both runs indicative of locomotor capacity of the lizard. The 0.15 m runs represent the first burst or escape response from a predator since the top velocity is usually reached in the first milliseconds of the response^58^ and represent the distance between two immediately contiguous shrubs. Meanwhile, the 1.05 m runs represent the longer distances lizards often use to activities such as foraging, territorial defence, escaping predators, and courtship, considering that in this population lizards run in general from one shrub to the other, which are 1 to 2 m apart (Fig. 4c).

The 0.15 m and 1.05 m running trials were carried out at eight temperatures: 12, 14, 18, 22, 24, 31, 35, 38 °C, included in the range of field active temperatures of *L. lineomaculatus* (10–40 °C^63^). Lizards were placed in a thermal chamber at stable temperatures for at least 30 min after equilibrium with target temperature before trials. We performed only two temperature trials per day, one in the morning and the other in the afternoon, leaving lizards enough time to rest between trials. Order of temperatures was haphazardly chosen for lizards (not following any particular randomization system), avoiding two contrasting temperatures (e.g. a very low temperature followed by a high temperature) on the same day, which could unnecessarily stress the lizards, following the methods of Angilletta et al.^83^, Fernández et al.^38^, Ibargüengoytía et al.^84^. Before each run, we measured the body temperature (*T*_b_) using the same methodology used for field T_b_.

Each lizard ran three consecutive times in each of the eight temperature trials, and then, we selected only the fastest non-stop run for the analyses.

We measured body mass before and after each trail using an Ohaus balance Scot Pro (± 0.01 g) and we did not find differences between them (Paired *t*-test, *t*_*1,37*_ = 0.711*, p* = 0.48 for Esquel individuals; *t*_*1,32*_ = 0.416*, p* = 0.68 for Calafate individuals). We considered the thermal tolerance breadth (TTB) as the difference between the critical thermal minimum (CTMin) and the critical thermal maximum (CTMax; methods for the estimation of CTMin and CTMax can be found in Supplementary Information on Materials and Methods) for each individual^85^.

### Environmental temperatures and potential lizard refuges

To measure environmental temperatures, we placed six models emulating a lizard’s shape in the temperate site (Esquel) and four models in the cold site (Calafate) connected by thermistors to data loggers (HOBO Temp H8, four-channel external data logger), between March 2017 and January 2018. The models were placed in potential refuges in which the species might seek temporary shelter (e.g., buried ~ 10–15 cm underground; beneath rocks; under tussocks) and in microenvironments outside of potential refuges (on the ground, under small bushes) partially exposed to environmental temperatures. At the site near Calafate, rocks suitable for refuging were very infrequent. This is relevant because rocks have been shown to be quite efficient as winter refuges in similar environments^36^, and as corridors and thermal buffers in low thermal quality environments^86^.

Temperatures were recorded every 30 min. The models were made of PVC pipe (1.5 × 8.0 cm section) which were then sealed at the ends with silicone (Fastix) to mimic body size, reflectance, thermodynamics, and shape of lizard’s bodies. We validated the models simultaneous temperature data from a live *Liolaemus lineomaculatus* individual and a model next to each other, exposing them to a sequence of temperatures. For the calibration, we used a heating lamp and a small terrarium, adjusting the model to mimic the position of the lizard (see Supplementary Fig. 1). Given that PVC models equilibrated too slowly with a live lizard during calibration to be considered representative of “operative temperature distributions” (sensu^87^), the term “operative temperatures” will not be employed in this study in relation to neither potential refuges nor the models set outside of potential refuges. Instead, we are considering the data as environmental temperatures recorded by data-loggers. After this calibration, we performed a regression between the model and the body temperature of the lizard (*T*_b_ = 2.82 + 0.912 × physical model; Adjusted R^2^ = 0.92; n = 2,510; Confidence Interval 0.88–0.94) and amended the values accordingly.

For the models’ data, we considered the active time for lizards as the period 09:00 to 19:00 h, using as reference the times of captures for the species from previous studies on *L. lineomaculatus*^63,88^. We discarded data from winter, given that lizards brumate during that season due to consistently low temperatures, snowfall and shorter days^88^. However, we included in the analyses data from the cold seasons of autumn and spring. We wanted to test whether lizards could run (or walk) during the infrequent warm days in autumn and spring, when temperature might allow for intermittent hours of activity.

In order to compare the “thermal quality” of potential refuges, we applied the concept of degree-days (sensu Lindsey and Newman^89^), using as reference the values of the mean CTMin for each location. Degree-days are the summation of temperature differences to a reference value over time. In this way, degree-days explain both the magnitude and duration that lizards would experience temperatures in relation to a reference chosen value. This metric allows a direct comparison of thermal regimes among different sites for many species or species populations^90–95^.

### Statistical analyses

We analysed the variability in body sizes and weights using body condition index (BCI), calculated as:

$${\text{BCI}} = {\text{M}}_{{\text{i}}} \;*\; \left[ {({\text{SVL}}_{0} ) / ({\text{SVL}}_{{\text{i}}} )} \right]^{{{\text{b}}_{{{\text{SMA}}}} }}$$

where *M*_*i*_ and *SVL*_*i*_ are the mass and SVL of the individual, *SVL*_*0*_ is the arithmetic mean SVL of the population, and b_SMA_ is the standardized major axis slope from the regression of ln body mass on ln SVL for the population (sensu Peig and Green^96^). The *b*_*SMA*_ exponent was calculated using the package ‘lmodel2’^97^ in R^98^.

Regarding 0.15 m runs and 1.05 m runs, we calculated the maximum speed achieved for each lizard (V_maxi_), the maximum speed achieved for the population (V_max_), and the thermal optimum (*T*_opt_), as the *T*_b_ at which speed is maximal for each individual. Additionally, we calculated the performance breadth (B_80_ and B_95_), the ranges of *T*_b_ at which performance is greater than or equal to 80% and 95% of the V_max_, respectively, following Hertz et al.^72^ and Angilletta et al.^83^ methodologies. Finally, we wanted to detect differences in performance considering only suboptimal temperatures (i.e., values below *T*_opt_), so we calculated maximum velocity at suboptimal temperatures (V_suboptimal_).

To estimate the V_maxi_, V_max_, B_80_ and B_95_ parameters for each population, we fitted a Generalized Additive Mixed-effects Model (GAMM) to the data obtained from the runs of all individuals using the “mcgv” package^99^. The GAMM approach^100^ allowed fitting the nonlinear relationship between temperature and speed with a smoother function, while also evaluating interindividual variability. We considered “individuals” (each lizard’s curve, obtained from all its temperature trials) as a grouping factor random effect, the BCI and sex as covariables, and the effect of temperature on speed as a fixed effect (one model for the 0.15 m runs and one for the 1.05 m runs). The model is further explained in the Supplementary Information on Materials and Methods.

Reported parameter estimates for both fixed and random effects were obtained with restricted maximum likelihood. All statistical analyses were performed with the R statistical software, version 3.5.3^98^ and the “mgcv” package, version 1.8-28^99^.

### Ethical statement

Captures were carried out with authorization from the Wildlife Service of the Province of Chubut (Permit # 0460/16 MP; Law XI N°10, Decree 686/90, Disposition #11/2016), signed by F. Bersano, Director of the Wildlife Service of the Province of Chubut, E-mail: direccionfaunayflorachubut@gmail.com. We followed the ASIH/HL/SSAR Guidelines for Use of Live Amphibians and Reptiles as well as the regulations detailed in Argentinean National Law #14,346.

## Supplementary information

Supplementary Data.

Supplementary Information.

## Data Availability

Data used for these analyses are available as a Supplementary Table and at Figshare (10.6084/m9.figshare.12857804).
