# Supplementary material for: Running performance with emphasis on low temperatures in a Patagonian lizard, Liolaemus lineomaculatus
Source: Sci Rep. 2020 Sep 7;10:14732. doi: 10.1038/s41598-020-71617-3 (PMC7477221; doi:10.1038/s41598-020-71617-3)
Supplement: Supplementary file 2 — Supplementary Information. [file 41598_2020_71617_MOESM2_ESM.pdf]

Running performance with emphasis on low temperatures in a Patagonian lizard, *Liolaemus lineomaculatus*.

Cecchetto, N. R.[a*]; Medina, S. M.[b]; Ibargüengoytía, N. R.[a]

[a] Instituto de Investigaciones en Biodiversidad y Medioambiente. Consejo Nacional de Investigaciones Científicas y Técnicas (INIBIOMA-CONICET), Quintral 1250, San Carlos de Bariloche, 8400, Argentina. Telephone: +54 9 294 442-3374. Emails: nrcecchetto@comahue-conicet.gob.ar; noraibarg@gmail.com.
[b] Centro de Investigación Esquel de Montaña y Estepa Patagónica. Consejo Nacional de Investigaciones Científicas y Técnicas (CIEMEP-CONICET), Esquel, Chubut, 9200, Argentina. Email: marlinmedina74@gmail.com. Telephone / Fax: +54 9 2945 453985
Correspondence to: Cecchetto at nrcecchetto@comahue-conicet.gob.ar; Telephone / Fax: +54 9 294-4423374

Supplementary Information

Supplementary information on Materials and Methods

Critical thermal minimum (CTMin)

In order to determine the critical thermal minimum (CTMin), we placed lizards individually in transparent plastic containers (15 × 10 × 5 cm) in an environmental chamber with adjustable cooling rates and a transparent door. Then, we lowered the temperature from room temperature (~20°C) to 0°C at a stable rate of 1°C / 5 min. To measure body temperature, we connected the lizards to a TC-08 Data Acquisition Module Omegas (8-channel USB thermocouple, ± 0.01° C) by an ultra-thin (0.08 mm) catheter thermocouple with measurements every second. We observed lizards throughout the experiment and, as soon as they started reaching values of ~7°C, the body temperature at which the individual was no longer able to right itself when placed on its back was recorded as CTMin.

Critical thermal maximum (CTMax)

In order to determine the panting threshold, we placed each lizard in an open-top terrarium (15 × 20 × 20 cm) with sand floor and an infrared 150-W lamp 40 cm overhead. We monitored the body temperature every second, as described for the CTMin observations. We carefully observed each lizard throughout the experiment. We considered the critical thermal maximum as the body temperature at which the individual was unable to right itself when it was placed on its back, starting to test as soon as individuals displayed the first signs of overheating (such as ventilating with the mouth open). The individuals were then immediately cooled down to allow recovery. All lizards survived and were in good conditions after the experiment.

Statistical analyses

For comparisons, this model incorporates a separate trend for each population. The interaction term in a GAM or GAMM allows testing the difference between the smooth estimated for the reference level and the nth level of the factor (in this case, the difference between the smoother for lizards from Esquel and the smoother for the ones from Calafate), following the methodology in Rose et al. (2012). For a more in-depth methodological explanation see Gavin Simpson's https://www.fromthebottomoftheheap.net/2017/12/14/difference-splines-ii/. The command *gam.check* in the 'mcgv' package was used to assess normality, homogeneity and model fit (Zuur et al., 2009).

In order to test the population trends at suboptimal temperatures we additionally fitted a GAMM to temperatures below T_{opt}, and the maximal speed at temperatures below the T_{opt} ($V_{\mathrm{suboptimal}}$), for SR and for LR.

We constructed a full model including all fixed and random effects. Then, we fitted the random effects using a restricted maximum likelihood approach and tested for the significance of random effects using likelihood ratio tests (LRTs). Subsequently, we fitted the fixed effects using the maximum likelihood approach, and comparison between models was performed using the Akaike Information Criterion (AIC).

Degree-days within thermal tolerance breadth

In this study, we chose the CTMin of each population as the reference value and then compared how often and how long *L. lineomaculatus* individuals would be above the species CTMin in their potential refuges. We used degree-days to compare among the different potential refuges, and calculated it using the formula:

$$\mathrm{DDTTB} = \sum_{i=1}^{n} \left| (\mathrm{T}i - \mathrm{TTB}_j) \ / \ 24 \right|$$

where *DDTTB* is potential refuge degree-day within each population's thermal tolerance breadth (TTB), and T_{i} refers to registered temperatures above CTMin and below CTMax for each *j* location (every hour).

Supplementary Table. AIC comparison of models with and without the mixed model structure for sprint-runs (SR), long-runs (LR) and SR and LR at suboptimal temperatures.

	0.15 m run	1.05 m run	Suboptimal 0.15 m run	Suboptimal 1.05 m run
AIC of models with mixed structure	195.33	-556.36	0.25	-380.37
AIC of models without mixed structure	246.30	-450.18	47.68	-300.39

Supplementary figures

Sup. Fig.1 Lizard PVC model calibration with a live *Liolaemus lineomaculatus* individual

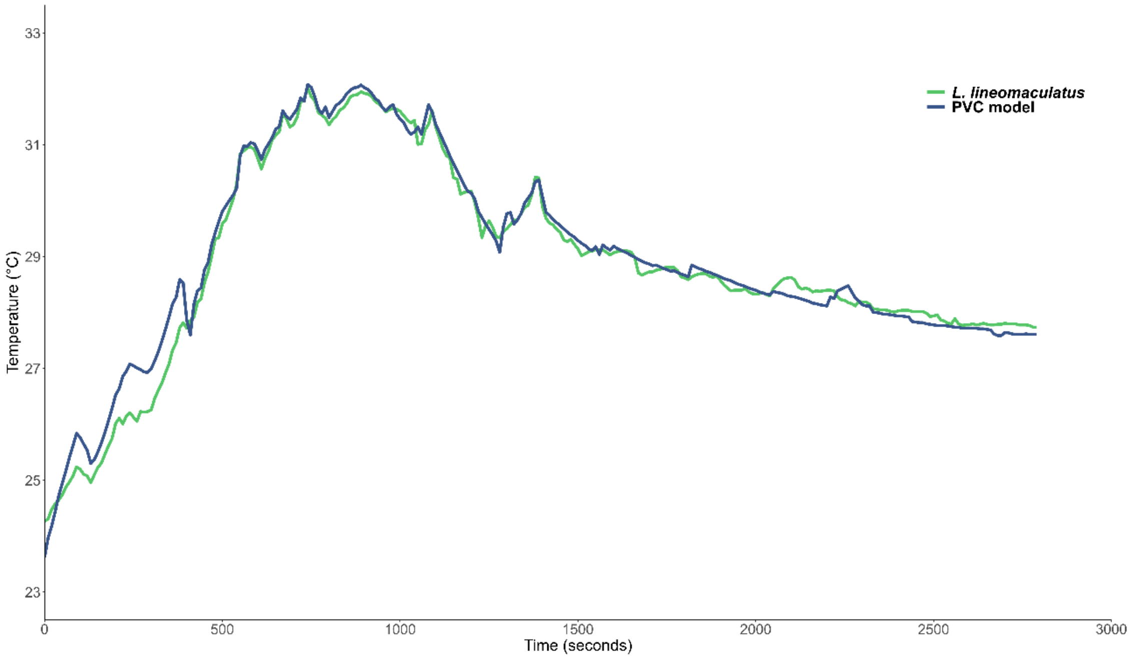

Sup. Fig.2 Individual sprint-runs (SR) of *Liolaemus lineomaculatus* individuals from Esquel from the Generalized Additive Mixed Model

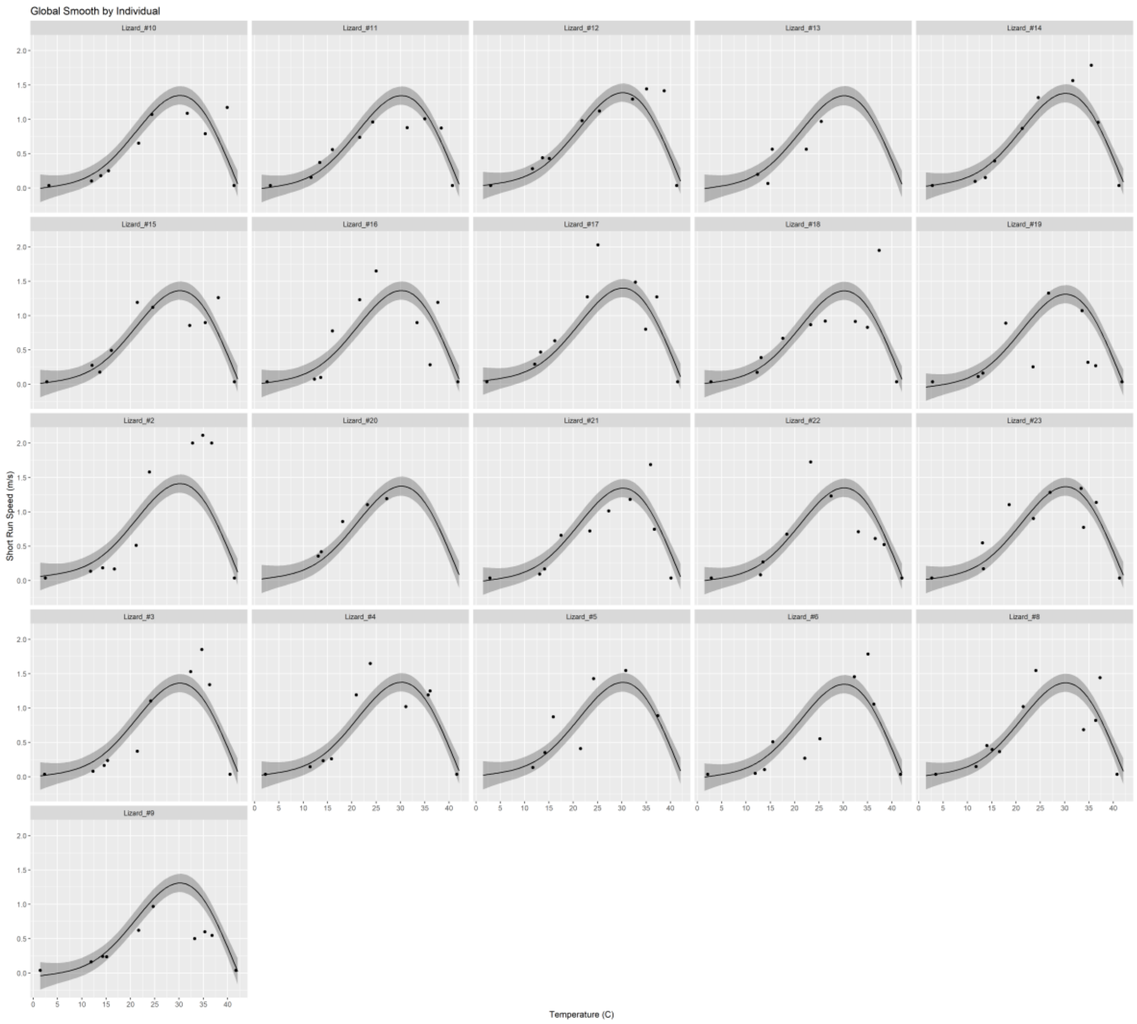

Sup. Fig.3 Individual long-runs (LR) of *Liolaemus lineomaculatus* individuals from Esquel from the Generalized Additive Mixed Model

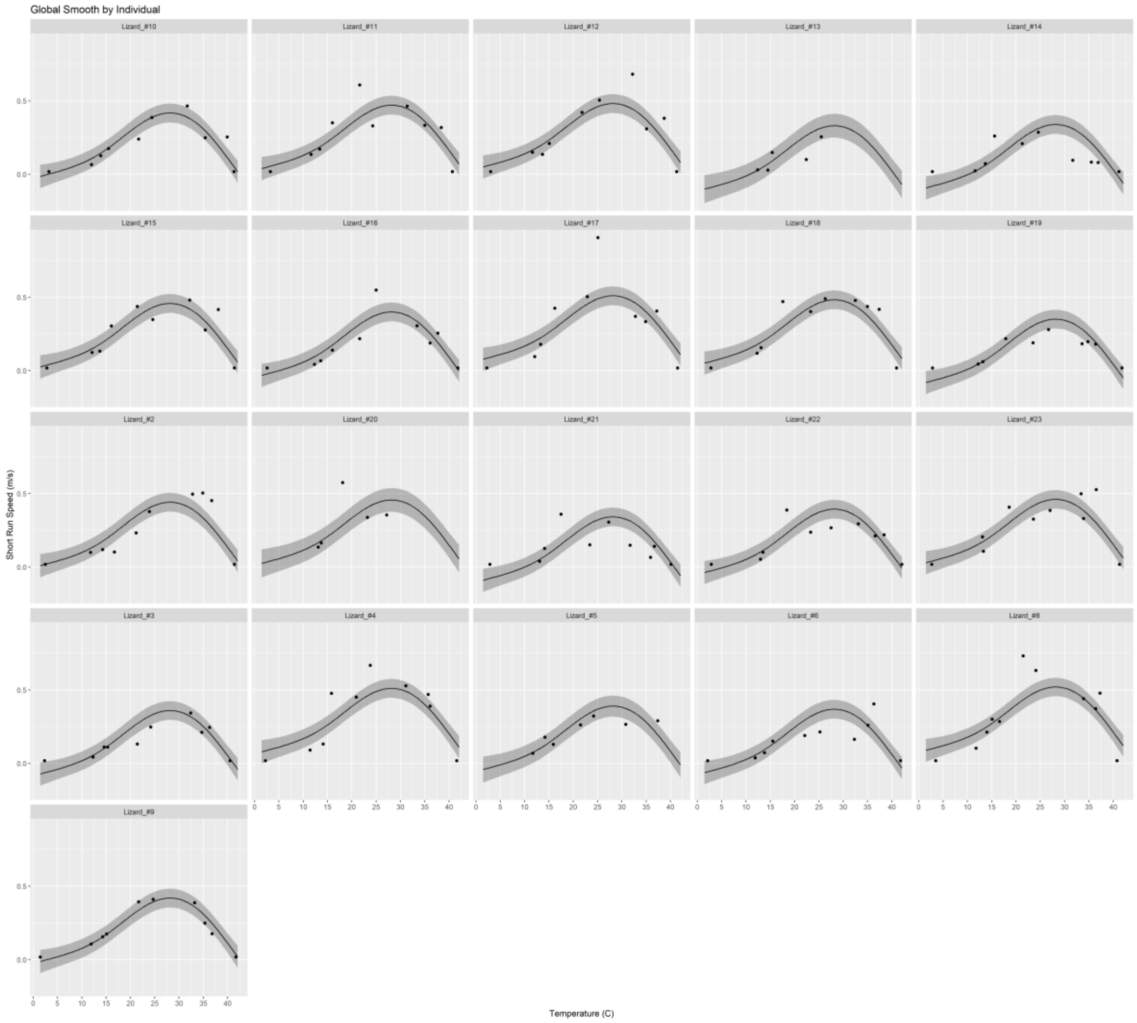

Sup. Fig.4 Individual sprint-runs (SR) of *Liolaemus lineomaculatus* individuals from Calafate from the Generalized Additive Mixed Model

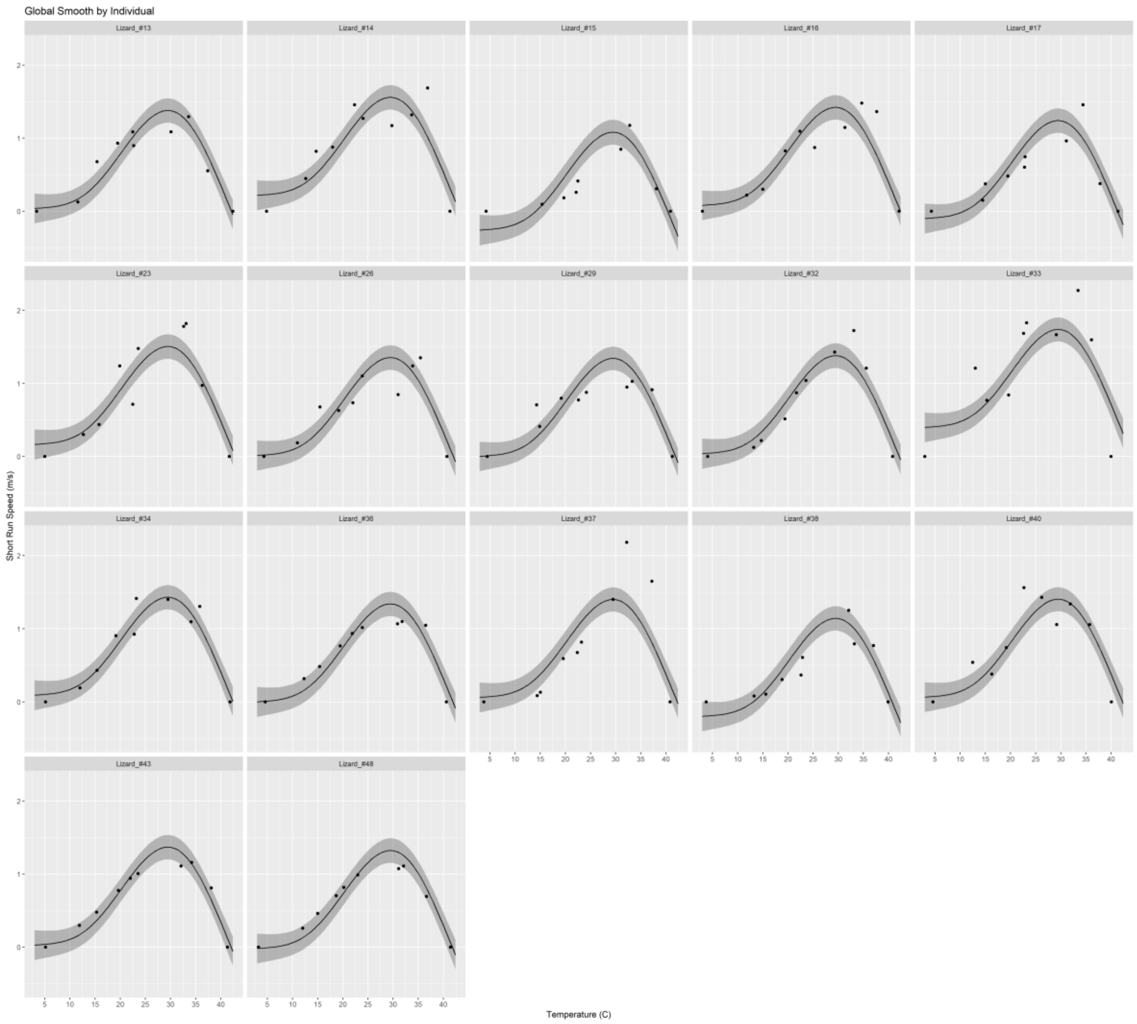

Sup. Fig.5 Individual long-runs (LR) of *Liolaemus lineomaculatus* individuals from Calafate from the Generalized Additive Mixed Model

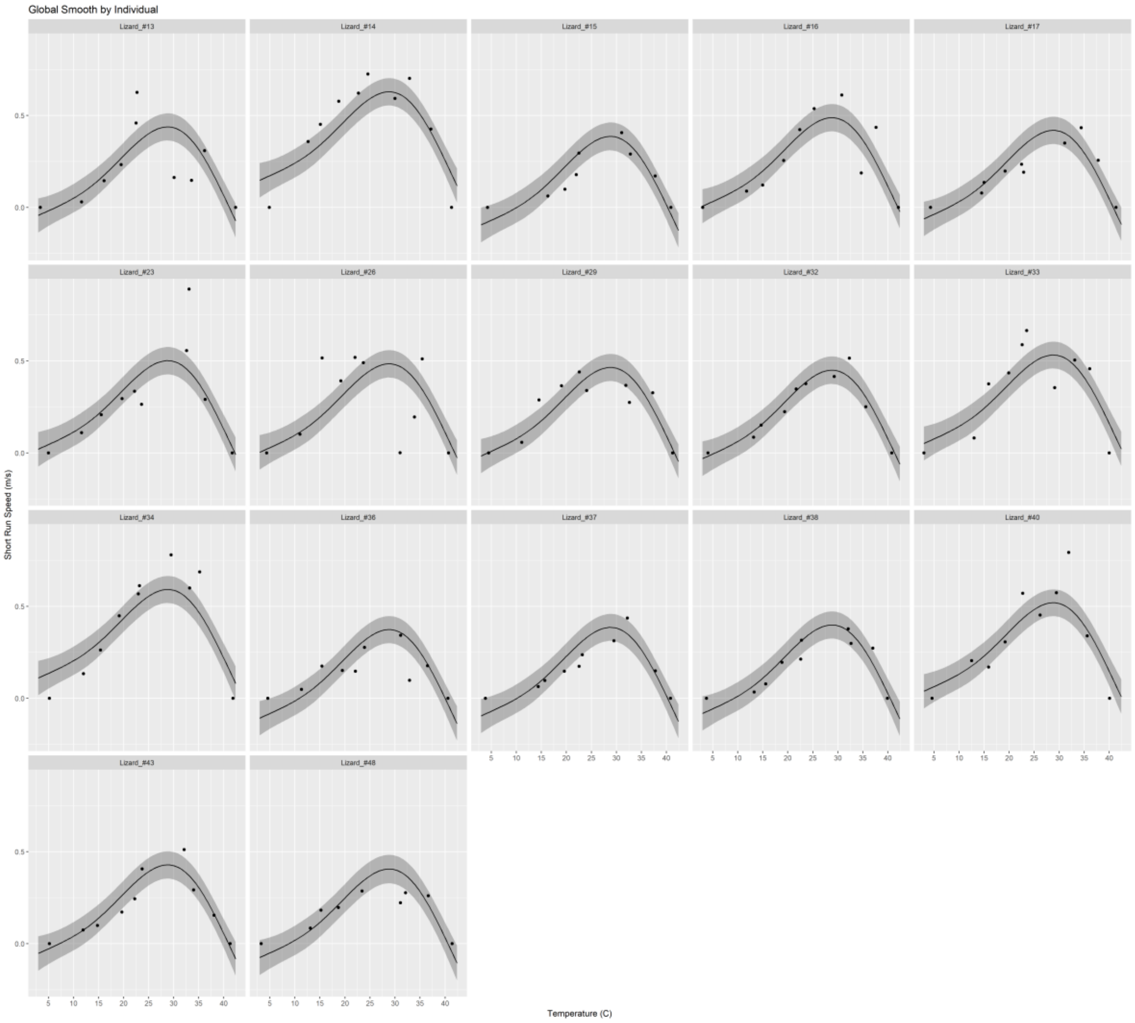

Literature Cited

Rose, N. L., Yang, H., Turner, S. D. & Simpson, G. L. An assessment of the mechanisms for the transfer of lead and mercury from atmospherically contaminated organic soils to lake sediments with particular reference to Scotland, UK. *Geochim. Cosmochim. Acta* **82**, 113–135 (2012).

Zuur, A., Ieno, E. N., Walker, N., Saveliev, A. A. & Smith, G. M. *Mixed effects models and extensions in ecology with R*. (Springer Science & Business Media, 2009).
